# The limits of privacy enhancement in healthcare federated learning: a ELSA perspective in the EU context

**DOI:** 10.3389/fdata.2026.1910002

**Published:** 2026-10-29

**Authors:** Marine Jacquemin

**Affiliations:** 1 Department of Radiation Oncology, GROW Research Institute for Oncology and Reproduction, Maastricht University Medical Centre+, Maastricht, Netherlands; 2 Department of Philosophy, Faculty of Arts and Social Sciences, Maastricht University, Maastricht, Netherlands

**Keywords:** bioethics, data ethics, data protection, digital health, federated learning, health data governance

## Abstract

Federated Learning (FL) usage is steadily increasing in healthcare. Offering an attractive alternative to centralized data processing, FL is often promoted as a privacy-enhancing technology (PET) because it keeps patient-level data local, exchanging only aggregated data or model updates. As such, it is frequently presented as a means of addressing the ethical, legal and societal challenges associated with the use of sensitive health data. This framing has contributed to growing policy interest in FL within initiatives such as the European Health Data Space (EHDS). This paper examines FL from an ethical, legal and societal (ELSA) perspective in the EU context, focusing on its interaction with the European data protection framework and contemporary debates on health data governance. First, it analyzes the interaction between FL architectures and the General Data Protection regulation (GDPR), showing that questions of controllership, joint responsibility, and identifiability persist despite decentralization, particularly in complex multi-institutional settings. Second, it argues that privacy-centric approaches to health data governance remain ethically insufficient because harmful inferences, discrimination, and collective harms may arise even when data remains formally anonymous. The dominant focus on reidentification risk thus reflects a narrow and individualistic conception of privacy that is poorly adapted to the contemporary data landscape of the EU. It fails to capture broader forms of harm, including group-level discrimination, inferential profiling, and structural power asymmetries. The paper concludes that, despite its framing as a PET, the use of FL alone is not sufficient to guarantee appropriate health data protection. Indeed, FL should also be approached as a distributed data-processing paradigm in which risks and benefits depend on implementation choices, institutional arrangements, and regulatory interpretation. Accordingly, FL should be situated within broader questions of data governance, including how access, accountability, and collaboration are structured and distributed across institutions.

## 1 Context

The potential of data-driven technologies to drive innovation in healthcare and provide better outcomes for patients is such that their application is rapidly expanding across various biomedical domains (Panayides et al., 2020; Dias and Torkamani, 2019; Perez-Pozuelo et al., 2020). To achieve optimal results, including increased precision and reduced risk of mistakes and bias, a large volume and range of data are needed. Bridging and combining different databases can significantly improve quality of research, especially regarding rare diseases, for which data contained in one single institution or nation is often insufficient to generate valuable and novel insights (Rieke et al., 2020). Recognizing this, the European Commission, together with member states, is actively working on implementing the European Health Dataspace (EHDS), which aims to foster cross-border health data exchange within the EU, thus enhancing health research and innovation (Fröhlich et al., 2025). Together with the AI Act, the EHDS forms part of the EU's emerging regulatory framework for trustworthy and interoperable health AI. As federated learning is increasingly promoted within this policy landscape, clarifying its legal and ethical implications becomes particularly important.

Indeed, while sharing health data can improve diagnoses accuracy and outcomes, it should not be pursued at all costs, ignoring ethical and legal concerns. Considering the sensitive nature of health data, along with the social stigma associated with certain conditions (Sartorius, 2007), it is important to patients that their health information does not end up in the wrong hands. This could lead to reduced opportunities (Stergiou-Kita et al., 2016), hiring discrimination (Brouwers, 2020) or difficulties in obtaining insurance or loans (Dumas et al., 2017).

To address these concerns, privacy-enhancing technologies (PETs), sometimes also referred to as “privacy-preserving” technologies (Kaissis et al., 2020), are increasingly implemented in biomedical research. PETs encompass a wide variety of technologies and techniques that aim to process data while maximizing data security and in compliance with data protection principles (Cho et al., 2024). Federated Learning (FL), a method allowing the generation of new knowledge or the training of AI models from multiple local data sources, is frequently cited as one of such techniques (Ali et al., 2023; Choudhury et al., 2020). Indeed, by eliminating the requirement to centralize all data by copying and/or transferring it to a single repository, FL reduces risks of data being illegitimately accessed, stolen or lost. Consequently, FL has been employed in multinational biomedical collaborations and large-scale European research initiatives (Frezza et al., 2024; Manthe et al., 2024; Hogenboom et al., 2025).

At the same time, the characterization of FL as having inherent privacy-enhancing properties deserves closer scrutiny. While often examined from a technical perspective, its ethical, legal and societal aspects (ELSA), including questions of accountability, fairness, transparency, and the distribution of benefits and risks, has received less attention.

This perspective paper critically examines the characterization of FL as a PET within biomedical research and European health data governance. It analyses FL under the EU General Data Protection Regulation (GDPR) and recent jurisprudence on pseudonymized data, before arguing that privacy-centered approaches overlook inferential and group-level harms. This paper argues that FL reduces certain forms of data exposure associated with centralized storage, but does not inherently prevent inferential, discriminatory, or governance-related harms. Building on this point, it criticizes the PET label attached to FL as flawed from both a legal and ethical perspective, as it risks conflating decentralized data processing with comprehensive patient protection.

## 2 Federated learning and privacy in data-driven biomedicine

FL is a new paradigm for knowledge generation and machine learning, which allows foregoing data centralization (Rieke et al., 2020). While data science enables the generation of new insights more rapidly and efficiently, it also requires access to substantial amounts of data. Traditionally, this has been achieved through copying and/or transferring data onto a single central database. Federated approaches, by contrast, do not necessitate transferring individual-level data. This has led to FL being advertised as privacy enhancing, which in that context is meant as reduced exposure of individual-level data and mitigation of adversarial attacks (Kairouz et al., 2021). As such, it presents an attractive way to reap the benefits of data analytics in biomedicine, where handling patient data ethically is of paramount importance. It can also facilitate access to health data that is fragmented across institutions and difficult to share across borders (Deimel et al., 2025). Federated architectures thus support broader European policy ambitions surrounding health data reuse and interoperability, offering an attractive compromise between supporting innovation and protecting patient information. Most notably, the EHDS, an initiative which seeks to facilitate cross-border data exchange and strengthen data-driven biomedical research within the EU, is currently testing a federated model for both primary and secondary health data usage (Kogut-Czarkowska and Shabani, 2025; Gonçalves et al., 2024). In the process, it further legitimizes it as a secure way to process sensitive data. In practice, FL deployments are sometimes combined with additional privacy-enhancing techniques such as secure aggregation, differential privacy, synthetic data, homomorphic encryption, secure multiparty computation or trusted execution environments (Kairouz et al., 2021). While these techniques differ substantially in their assumptions and guarantees, differential privacy (DP) is distinctive in that it provides a formal mathematical framework for quantifying and limiting privacy loss during model training. By contrast, FL itself offers no comparable guarantee (Truong et al., 2021). As a result, recent reviews have suggested that FL may only provide sufficient privacy guarantees for medical research when employed in combination with both DP and secure multiparty computation (Brauneck et al., 2023). Many of the privacy assurances associated with FL thus derive from supplementary safeguards rather than from decentralization alone. Furthermore, implementation choices also substantially influence the extent to which these safeguards mitigate privacy risks in FL. For example, decisions regarding the selection and configuration of DP mechanisms, the design of aggregation procedures, and the governance of model or data access all influence the balance between privacy protection and utility for research or model training (Truong et al., 2021; Brauneck et al., 2023). Similarly, in healthcare settings, decisions regarding data standardization and harmonization, as well as the implementation of minimum sample size requirements for analyses, all influence which datasets can meaningfully participate in FL and may influence the representativeness of resulting models, particularly in contexts involving rare diseases or small patient populations (Rieke et al., 2020). Consequently, the privacy benefits of FL cannot be assessed independently from the technical and institutional choices through which federated infrastructures are implemented.

## 3 Federated learning and privacy in the European data protection framework

Assessing the interaction of FL and privacy under EU law requires distinguishing between three separate questions: (1) which actors bear legal responsibility within federated infrastructures, (2) whether pseudonymized data remain personal data within the meaning of the GDPR, and (3) which legal obligations are altered, or left unchanged, within decentralized architectures. This analytical distinction reflects the questions that have emerged in the legal literature on FL, which has focused in particular on the GDPR status of FL outputs, the allocation of controller roles, and the impact of supplementary privacy-enhancing technologies on legal compliance (Brauneck et al., 2023). While FL changes the technical organization of data processing, it does not automatically eliminate questions of controllership, identifiability, or accountability.

Let us first examine the distribution of responsibility in FL infrastructures. The main stakeholders involved in a typical federated learning infrastructure, together with their potential roles under the GDPR and corresponding responsibilities, are summarized in Table 1.

**Table 1:** Stakeholders roles and responsibilities in federated data sharing.

Actor	Role in FL	GDPR role	Key responsibilities
Patient	Provide personal data processed within the federated infrastructure	Data subject	Limited. May exercise rights under the GDPR (access, rectification, objection…) where applicable
Hospitals or other health institutions	Function as local nodes. Store patient data locally and perform model training or data analysis on local datasets	Controller or joint controller	Lawful processing of patient data, implementing appropriate safeguards, and facilitating data subject rights
Central aggregator	Coordinates learning and aggregates model updates or local results	Controller, joint controller, and/or processor	Coordinates model training and aggregation. Depending on governance arrangements, may also share responsibility for security, transparency and accountability
Research consortium	Defines research objectives and technical architecture	Controller or joint controller	Defines research objectives and governance arrangements. May share responsibility for accountability, risk assessment and selection of appropriate safeguards.
Cloud and/or infrastructure provider	Provides and hosts software and/or computational infrastructure	Processor	Implements appropriate security measures and safeguards, and processes data according to documented instructions (art. 28 GDPR)

Precise GDPR role determination (processor vs. controller vs. joint controller) depends on the allocation of decision-making authority and cannot be inferred solely from the technical architecture but must instead be assessed on a case-by-case basis.

Technical discussions surrounding FL frequently imply that because patient data remains locally stored within participating institutions, legal responsibility similarly remains local. Under the GDPR, however, data decentralization does not automatically equate to decentralization of controllership. A thorough analysis requires examining not only where data is stored, but also which actors determine the purposes and means of processing that data [art. 4(7) GDPR]. Within the GDPR framework, these actors qualify as data controllers and remain subject to the obligations associated with that role [art. 4(7)]. Identifying which participants qualify as data controller under the GDPR is crucial. Indeed, they are responsible for ensuring that data processing is conducted in accordance with data protection regulations [art. 24(1) GDPR].

Federated biomedical infrastructures often involve a wide range of participants, including hospitals, research institutions, cloud platforms, service providers, and external model developers. Local healthcare institutions may continue to act as controllers with respect to the patient data they hold. However, the distributed and often collaborative nature of FL usually entails that more than one actor is involved in deciding the purpose and means of processing. Healthcare institutions may determine these jointly, together with the entities coordinating the federated infrastructure, involved research consortia, or central aggregators. In this case, they may all qualify as joint controllers (art. 26 GDPR). Depending on their involvement in defining research objectives or the operation of the federated system, data users or external model developers may likewise qualify as joint controllers rather than merely recipients of data or processors.

In multinational FL projects, responsibility allocation can therefore become complex, particularly where multiple actors collectively participate in determining data access conditions and procedures. This means that, rather than eliminating governance obligations, federated architectures redistribute them across several interconnected actors.

This has important practical implications. Determining which entity bears responsibility for transparency obligations, security measures, or responses to data subject requests may become more difficult within decentralized infrastructures involving numerous institutional and technical intermediaries (European Data Protection Board, 2020). Consequently, the deployment of FL in practice is not a solely technical matter: it also introduces a new governance model requiring careful allocation of responsibilities between participating actors.

A second important point is to assess the interaction of FL and privacy from the perspective of EU data protection law. Although FL is frequently presented as a privacy-enhancing technique because personal data remains local, its legal implications must be assessed under the GDPR. The framework of data protection outlined in the GDPR revolves around the privacy-adjacent concept of *personal data*, namely information relating to an identified or identifiable natural person [art. 4(1)]. As this is the subset of data that the regulation aims to protect, understanding what constitutes *personal* data becomes central to determining whether data processing activities fall within the scope of EU data protection law.

Under EU data protection law, only data that has been anonymized, escapes the definition of personal data (recital 26 GDPR; art. 29 Working Party). By contrast, pseudonymized data, remains personal data because they can still be attributed to identifiable individuals with the use of additional information (art. 4(5) GDPR). In practice, pseudonymization often involves removing or obscuring identifiable components, such as names and addresses, so “it can no longer be attributed to a specific data subject without the use of additional information” [art. 4(1)] while retaining a mechanism that allows the data to be linked back to a specific individual when necessary. In biomedical contexts, complete anonymization is often impractical, because patient records must remain linkable to individuals to ensure continuity of care, record updates, and patients' access to their own information. As a result, health data is usually kept pseudonymized, as a compromise between usability and security.

The distinction between anonymization and pseudonymization is not always straightforward. Recital 26 GDPR adopts a contextual approach to determining whether a dataset can be considered anonymous, requiring consideration of all the means *reasonably likely* to be used to identify individuals. This makes anonymization highly context-dependent, as implementation settings and technological developments continuously alter what may count as reasonably identifiable.

Recent jurisprudence from the Court of Justice of the European Union clarified that pseudonymized data may not constitute personal data in all contexts (Court of Justice of the European Union, 2025). Instead, identifiability also depends on whether the actor processing the data has access to the additional information necessary for reidentification.

This distinction is particularly relevant for FL architectures, where data remain distributed across participating institutions while computational tasks are performed locally. Let us consider a common implementation scenario involving health data: the Personal Health Train (PHT) model (Beyan et al., 2020). In the PHT, multiple institutions make their datasets available through “data stations,” which function as federated nodes within the system. Rather than transferring patient data to a central repository, an FL algorithm is deployed across these stations, where each node performs data analysis or local model training on its own dataset. The resulting statistical results or model model updates are then aggregated into a final output. In this scenario, two distinct operations can be identified: (a) the local processing of health data within each node and generation of partial results or model updates and their aggregation, and (b) subsequent sharing and use of the final aggregated result or model.

At the level of the data stations or federated nodes (a), the legal qualification of the data depends primarily on whether the information remains personal data within the meaning of the GDPR, which is not always straightforward. In many FL deployments involving health data, local institutions retain control over reidentification keys while computations are performed locally and by another party within decentralized nodes. Whether such processing falls within the scope of the GDPR therefore depends largely on whether the actor processing the pseudonymized datasets—the controller—has reasonable access to the additional information necessary for reidentification (recital 26). Where reidentification keys remain available to data controllers, the information generally continues to qualify as personal data under article 4(1). It is only when they are not that pseudonymized data may fall outside GDPR scope: recent reviewers have argued that local models and model updates will often continue to qualify as personal data under the GDPR, while the legal status of aggregated global models remains considerably less certain (Brauneck et al., 2023).

However, as discussed earlier, the distributed and collaborative nature of FL implementation settings, leads to controllership often being joined between multiple actors, making interpreting that question complex. It is even more so in biomedical contexts, where health data is recognized as highly sensitive (art. 9), rich in contextual information, highly granular, and potentially identifiable, especially in the context of rare diseases or small patient populations. In such circumstances, cautious interpretation of these dispositions by the (joint) controllers remains the most advisable path forward, not only to avoid legal repercussions, but also out of respect for patients. Even when deploying FL, the full range of GDPR obligations, including lawful processing requirements, accountability obligations, and the completion of Data Protection Impact Assessments (DPIAs), are likely to remain applicable in most cases.

Residual risks also persist at the level of model or data aggregation and output sharing (b). Technical and human failures can occur, and so can malicious attacks. Although federated systems generally exchange model parameters or statistical summaries, which are often assumed to significantly reduce identifiability risks, in certain cases, model outputs (Liu et al., 2022) or gradients (Boenisch et al., 2023; Huang et al., 2021) have been shown to carry residual information about training data. This is particularly the case in settings involving small datasets or rare diseases, where patients cannot “hide in numbers” and thus aggregation methods are less effective. This paper does not aim to provide a comprehensive list of all potential privacy risks within FL infrastructures. However, the documented existence of these risks shows that while FL may enhance privacy by reducing certain forms of exposure associated with centralized databases, it does not eliminate obligations relating to lawful processing, accountability, proportionality, or risk mitigation (Brauneck et al., 2023).

Consequently, DPIAs and controllership agreements remain important within federated biomedical infrastructures. DPIAs in particular are mandated by the GDPR in cases where processing is likely to result in a high risk for individuals [art. 35(1)] as well as when processing large quantities of sensitive data [art. 35(2)]. Beyond legal compliance, they function as structured reflection tools through which institutions identify residual risks, document safeguards, clarify governance responsibilities, and evaluate whether technical protections are proportionate to the sensitivity of the data involved. GDPR applicability in federated systems therefore depends less on decentralization than on the interaction between technical safeguards, institutional governance arrangements, and the residual identifiability of outputs. Aggregation and decentralization may reduce certain risks, but do not inherently eliminate data protection obligations. Furthermore, when AI systems trained using FL fall within the scope of the AI Act (art. 2–3), compliance with the GDPR alone does not exhaust the applicable regulatory framework. Depending on the system and its intended use, additional obligations concerning risk management, data governance, transparency, and human oversight may also arise under the AI Act (art. 9–10; 13–14).

## 4 Ethical limits of privacy-centered governance of health data

The discussion above shows that FL does not eliminate data protection obligations but rather redistributes them across decentralized infrastructures. Furthermore, assessing FL solely through the lens of the GDPR also has its limitations. Because the current European data protection framework is primarily concerned with individual privacy and identifiability, it does not necessarily capture all of the ethical issues that may arise from health data processing. The following section therefore considers the broader ethical limits of this privacy-centered approach to data governance.

Even where FL systems satisfy legal data protection requirements, important ethical concerns that extend beyond the GDPR's primary focus on individual privacy and reidentification risks remain. Indeed, harmful consequences can arise even when data remain anonymized, pseudonymized, or otherwise formally “private.” An exclusive focus on individual-privacy can create the illusion that, when employing anonymization techniques, data can be used freely without causing harm. Yet, harms deriving from data processing aren't limited to individual privacy breaches: in a world where systems trained on our data are increasingly employed to calculate insurance premiums or train medical AI, limiting risk assessments to individual reidentification risks is short-sighted (Cofone, 2023).

Indeed, even when no individual patient is directly identifiable, their data can contribute to biased AI models or statistical results that may cause harm (Mittelstadt and Floridi, 2016). For example, bias within AI systems employed in clinical settings have led to situations where patients of color were consistently deprioritized compared to white patients (Obermeyer et al., 2019; Cross et al., 2024). More generally, statistical inferences have been shown to cause group harms by negatively impacting people belonging to minority groups in domains such as genomics and data-centric biomedical research (Chapman et al., 2025). These harms can materialize in the shape of discriminatory decision-making, profiling, reinforcement of existing inequities, and the exclusion of minority health needs from research design and clinical practice (Mittelstadt and Floridi, 2016; Eitenberger et al., 2025).

The fact that most data protection frameworks remain strongly centered on individual conceptions of privacy is thus concerning. Such approaches were developed before modern data analytics, predictive modeling, and algorithmic governance. Today, as harms frequently emerge not from disclosure of personal information itself but from the ability to derive sensitive patterns, correlations, and predictions from large datasets, data governance frameworks are increasingly challenged (Cofone, 2023).

Emerging frameworks attempt to bridge that gap by proposing new ways to govern data. Among them, data solidarity (Prainsack et al., 2022) emphasizes collective responsibilities and benefits arising from data use, and data justice (Braun and Hummel, 2022) brings attention to the way that data practices distribute power, burdens, and opportunities across society. These approaches call for consideration of the large power assymetries existing between data subjects and data controllers. They also encourage thinking in terms of group harms and benefits, rather than individual rights. Finally, they advocate moving beyond outdated binaries of personal vs. non-personal, health vs. non-health, or sensitive vs. non-sensitive data. As technology and data practices evolve, these categories have become obsolete: data is often bundled or linked, such that seemingly innocuous data can often be traced back to individuals. For example, smartwatch and location data were found to be easily reidentifiable even when employing anonymization techniques (Lange et al., 2025; de Montjoye et al., 2013; Sharon, 2021).

A second, connected ethical issue with the overwhelming focus on privacy as a marker of ethical health data governance is that it tends to obscure other ethical concerns. Scholars have frequently pointed out that privacy, which is often conflated with security, is seen as the most solvable and concrete issue within data-driven practices (Sharon, 2021; van Daalen et al., 2025). The popularity of PETs and other “privacy by design” methods are testament to that. But responsible governance of medical FL systems cannot be limited to minimizing the exchange of identifiable patient data. A sole focus on individual reidentification risks is not only counterproductive; it also has a pervasive effect. It is used to legitimize the actions of data controllers even when their activities merit further ethical scrutiny, and it takes time and attention away from other ethical concerns, such as sphere transgressions or contextual integrity (Sharon, 2021; Nissenbaum, 2004), where violations of privacy are defined based on context and actors involved, a relativity which isn't easily translated within data protection frameworks. Other important ethical endeavors are also comparatively neglected. such as the inclusion of minority groups and meaningful patient participation. Careful assessment of research questions and their outputs is also needed, not only for security risks, but also for their anticipated harm or benefit to the public (El-Sayed et al., 2025).

It is true that the decentralized data analysis or model training aspect of FL does offer some additional security with regards to centralized alternatives, by virtue of keeping individual-level data local. From that perspective, FL does offer some improvement which some may consider sufficient to claim that it is privacy enhancing, especially when coupled with supplementary technologies (such as DP) and effective governance mechanisms (Alvarenga et al., 2026). Whether FL truly merits the PET label is not so much the topic of this article; rather, it critically examines what this label does from an ELSA perspective. Indeed, positioning FL as a PET risks reinforcing a narrow understanding of what ethical health data governance entails, by, in this case, framing it primarily in terms of limiting patient-level data circulation,. The fact that much of the discourse around FL focuses on these privacy claims diverts attention away from research into harvesting its technical specificities—most notably decentralization of data and opportunities for distributed decision-making—for more ethical data governance. This could mean, for example, exploring better consent management mechanisms and increasing transparency for both local institutions and patients. Another worthwhile and underexplored area of research would also be to determine if and how FL implementation mayimprove data sovereignty, not only in the sense of national governance, but also for individuals who increasingly feel that they have lost control over the use of their data (Braun et al., 2021).

## 5 Conclusion

FL offers promising new ways in which to process patient data and generate valuable biomedical insights while reducing the risks associated with centralized data pooling (Rieke et al., 2020). In the context of European initiatives such as the EHDS, the use of FL may facilitate cross-border collaboration and support the expansion of data-driven health research (Gonçalves et al., 2024).

However, while generally more secure than its centralized counterparts, FL does not inherently guarantee complete privacy or patient anonymity (Truong et al., 2021). While it can reduce certain risks associated with centralized data storage, it does not eliminate risks related to inference, model leakage, governance challenges, or downstream misuse of outputs (Liu et al., 2022; Boenisch et al., 2023; Huang et al., 2021). In all data-sharing infrastructures, privacy protection remains contextual, probabilistic, and dependent on implementation choices rather than being guaranteed by technical architecture alone.

Against this background, the PET label risks overstating what federated systems can provide, both from a technical and normative perspective. It not only suggests stronger guarantees than federated systems can realistically provide but also contributes to a narrow understanding of what constitutes harm, by limiting it to reidentification risks. Therefore, this paper argues that framing FL primarily as a PET obscures its broader role in introducing a socio-technical framework in which legal responsibilities, governance structure, and ethical considerations are equally central to trustworthy biomedical research.

The value of FL instead lies not in resolving privacy concerns *per se*, but in reshaping how data access, collaboration, and accountability can be structured across institutions in federated architectures. In practice, FL projects should therefore assess not only technical performance, but also the allocation of controllership responsibilities, the adequacy of supplementary PETs, and the potential impacts of model outputs on populations, particularly vulnerable or underrepresented patient groups. Exploring these avenues requires sustained attention to governance arrangements, and transparency and accountability mechanisms, alongside technical safeguards. Such mechanisms may include structured ethical oversight for the approval and monitoring of federated analyses through the establishment of dedicated governance or ethics committees. Federated infrastructures and algorithms would also benefit from being audited for technical robustness, legal compliance and accountability mechanisms by independent third partie. Finally, joint controllership and data-sharing agreements should be tailored to the distributed nature of FL and clearly define the rights and responsibilities of participating stakeholders.

Reframing FL in this way shifts attention toward opportunities that are often overshadowed by privacy-centric discourse: improved control over data, more meaningful consent management, and greater data sovereignty for both local participating institutions and individual patients. A more adequate regulatory and ethical framework would therefore move beyond the narrow question of whether personal data is exposed and instead assess how federated infrastructures distribute responsibility, power, risks, and benefits among institutions, patients, and populations.

The central question is therefore not whether FL preserves privacy, but rather under which technical, legal, and governance conditions it can contribute to trustworthy and socially beneficial biomedical research. Adressing this question requires future FL initiatives to integrate legal and ethical expertise alongside technical development, establish appropriate governance structures before data sharing begins, and continuously assess the broader implications of model outputs throughout the lifecycle of federated projects. By extending evaluation beyond privacy alone to encompass legal, ethical, and governance considerations, FL can better realize its potential as a facilitator of collaborative and responsible biomedical research.

## Data Availability

The original contributions presented in the study are included in the article/supplementary material, further inquiries can be directed to the corresponding author.
